# Establishing a Statistical Link between Network Oscillations and Neural Synchrony

**DOI:** 10.1371/journal.pcbi.1004549

**Published:** 2015-10-14

**Authors:** Pengcheng Zhou, Shawn D. Burton, Adam C. Snyder, Matthew A. Smith, Nathaniel N. Urban, Robert E. Kass

**Affiliations:** 1 Program for Neural Computation, Carnegie Mellon University, Pittsburgh, Pennsylvania, United States of America; 2 Center for the Neural Basis of Cognition, Pittsburgh, Pennsylvania, United States of America; 3 Department of Biological Sciences, Carnegie Mellon University, Pittsburgh, Pennsylvania, United States of America; 4 Department of Ophthalmology, University of Pittsburgh, Pittsburgh, Pennsylvania, United States of America; 5 Department of Neurobiology, University of Pittsburgh, Pittsburgh, Pennsylvania, United States of America; 6 Department of Statistics, Carnegie Mellon University, Pittsburgh, Pennsylvania, United States of America; 7 Department of Machine Learning, Carnegie Mellon University, Pittsburgh, Pennsylvania, United States of America; Indiana University, UNITED STATES

## Abstract

Pairs of active neurons frequently fire action potentials or “spikes” nearly synchronously (i.e., within 5 ms of each other). This spike synchrony may occur by chance, based solely on the neurons’ fluctuating firing patterns, or it may occur too frequently to be explicable by chance alone. When spike synchrony above chances levels is present, it may subserve computation for a specific cognitive process, or it could be an irrelevant byproduct of such computation. Either way, spike synchrony is a feature of neural data that should be explained. A point process regression framework has been developed previously for this purpose, using generalized linear models (GLMs). In this framework, the observed number of synchronous spikes is compared to the number predicted by chance under varying assumptions about the factors that affect each of the individual neuron’s firing-rate functions. An important possible source of spike synchrony is network-wide oscillations, which may provide an essential mechanism of network information flow. To establish the statistical link between spike synchrony and network-wide oscillations, we have integrated oscillatory field potentials into our point process regression framework. We first extended a previously-published model of spike-field association and showed that we could recover phase relationships between oscillatory field potentials and firing rates. We then used this new framework to demonstrate the statistical relationship between oscillatory field potentials and spike synchrony in: 1) simulated neurons, 2) *in vitro* recordings of hippocampal CA1 pyramidal cells, and 3) *in vivo* recordings of neocortical V4 neurons. Our results provide a rigorous method for establishing a statistical link between network oscillations and neural synchrony.

## Author Summary

Spike synchrony, which is widely reported in neural systems, may contribute to information transmission within and across brain regions. Critical to this theory is the potential link between oscillatory activity and synchronous spiking. In this article we provide a method for establishing the statistical association of spike synchrony with an oscillatory local field potential. We demonstrate the value of this technique by numerical simulation together with application to both *in vitro* and *in vivo* neural recordings.

## Introduction

A leading theory of current neuroscience is that synchronous firing of neurons driven by network-wide oscillations may encode and transmit information within and across brain regions [1–9]. Supporting this theory, a number of studies have suggested that synchronous firing of action potentials or “spikes” may indeed occur in conjunction with oscillations in local field potential (LFP) [10–14]. However, a missing link in this theory has been the ability to dissociate enhanced spike synchrony due to network-wide oscillations from enhanced spike synchrony that may be due to other measured or unmeasured sources. Recently, we developed a statistical framework in which the association between spike synchrony and measured covariates may be assessed [15, 16]. Here we show how this approach may be applied to describe the relationship between spike synchrony and oscillatory activity.

Using point process regression models, which take the form of generalized linear models (GLMs), our statistical framework compares the observed number of synchronous spikes within a small time window (here, 5 ms) to the number predicted by chance, under varying assumptions about the factors that affect the firing of each individual neuron [15, 16]. The number of synchronous spikes predicted “by chance” refers here to the number predicted under conditional independence after conditioning on the various measured factors that have been hypothesized to affect individual-neuron spiking. For example, two neurons having fluctuating stimulus-driven firing rates will produce some number of synchronous spikes even if they are acting independently. The point process regression method fits fluctuating firing rate functions for each neuron separately, then predicts the number of synchronous spikes under conditional independence (i.e., after conditioning on these fluctuating firing rates), and compares the prediction to the observed number of synchronous spikes. In this way, a single factor may be either included or excluded from the regression model in order to quantify that factor’s ability to explain the observed spike synchrony.

In this article, we consider the contribution of network-wide oscillations by comparing observed and predicted spike synchrony after conditioning on the phase of an LFP representing a network-wide oscillation. Thus, we predict spike synchrony with and without inclusion of LFP phase as an explanatory variable for each neuron separately. To demonstrate that increased spike synchrony is associated with a network-wide oscillation, we would begin by establishing that, without considering LFP phase, the observed number of synchronous spikes is greater than the predicted number by a statistically significant magnitude, after conditioning on both stimulus-driven firing rates and recent post-spike history effects. This would indicate a failure of the phase-free model to accurately account for spike synchrony. We would then include the LFP phase in the model, and if it succeeded in predicting spike synchrony, then we would conclude that LFP phase can explain the remaining spike synchrony. Furthermore, we could estimate the proportion of excess synchronous spikes accounted for by the LFP phase. The same procedure could be used, instead to demonstrate the role of network-wide oscillations in suppressing spike synchrony.

In order to carry out this general procedure, we first need to model an individual neuron’s spiking probability in terms of LFP phase. We follow [17], which recently described and assessed point process regression models that include a sinusoidal phase term. We enhance their approach by weakening the sinusoidal assumption, allowing the phase relationship to be nonparametric as in [18], and we add to the favorable results of [17] by showing that, in estimating phase relationships, the point process regression model can reduce bias and mean-squared error in comparison with the more familiar spike phase histogram approach. Using this point process regression model, we are then able to quantify the dependence of synchronous spiking on an oscillatory modulation. We illustrate the method using simulated neurons, *in vitro* recordings of hippocampal CA1 pyramidal cells, and *in vivo* recordings of neocortical V4 neurons from a behaving monkey.

## Results

### Point Process Model for Spike Trains

We assume that the spiking of each neuron follows a point process and, following [19] (page 592), we write its conditional intensity function as *λ*(*t*∣*H*_*t*_,*X*_*t*_), where *H*_*t*_ represents the spike history (auto-history), and the covariate *X*_*t*_ represents other external factors. In this work, we let *X*_*t*_ include the stimulus and the LFP phase, denoted by *X*_*t*_ = (*S*_*t*_,Φ_*t*_). We assume the conditional intensity takes a multiplicative form, which becomes additive on the log scale:

$$\begin{array}{cc} \log\lambda (t|H_{t},X_{t}) & =f_{1}\left(S_{t}\right)+f_{2}\left(H_{t}\right)+f_{3}\left(\Phi_{t}\right)\\ & =\log\lambda_{1}\left(t\right)+\log\lambda_{2}\left(t-t^{*}\right)+\log\lambda_{3}\left(\Phi_{t}\right) \end{array}$$ (1)

where t* is the last spike time preceding *t* (see Materials and Methods).

We use splines to capture stimulus and auto-history effects, and circular splines to capture LFP phase effects. Our point process model thus takes the form of a standard generalized linear model (GLM). We also ensure identifiability by imposing a set of restrictions (Eqs (20) and (21)), which are implemented within a maximum likelihood estimation (MLE) algorithm. The parametric bootstrap is used for acquiring 95% confidence bands.

**Fig 1:**
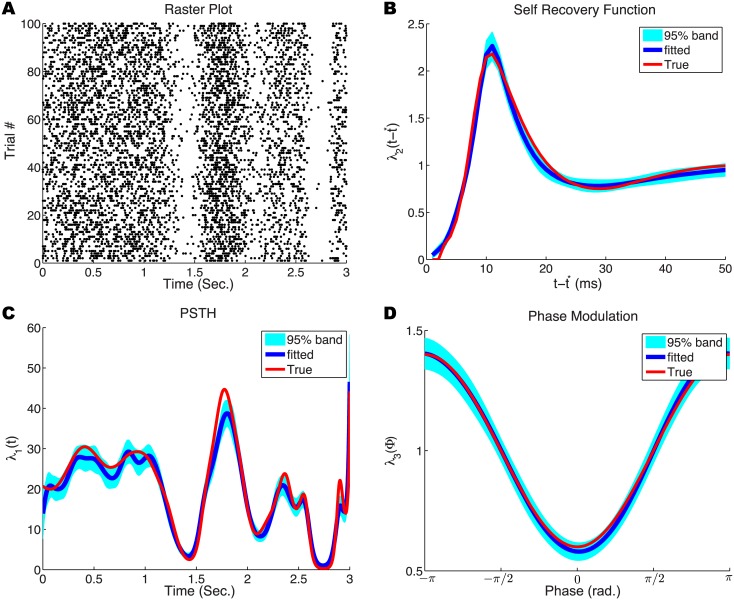
Simulated spike trains and results of model fitting. (A) Simulated spike trains in response to a fluctuating stimulus and oscillatory drive. (B,C,D) Ground truth (red) and fitted results (blue) for different terms in the firing-rate probability model. For each fitted result, we used a parametric bootstrap to determine the 95% confidence band (cyan). (B) Effect of auto-history *λ*_2_(*t* − t*) on output firing rate. (C) Effect of stimulus *λ*_1_(*t*) on output firing rate. (D) Phase modulation curve *λ*_3_(*ϕ*) of firing rate.

To illustrate the ability of the MLE algorithm to recover the model in Eq (1), we simulated 100 spike trains (Fig 1A) with known functions *λ*_1_(*t*), *λ*_2_(*t* − t*) and *λ*_3_(*ϕ*). Using the simulated spike trains and phase of the oscillatory drive (representing a network-wide oscillation), the MLE algorithm accurately fit the underlying spike history (Fig 1B), stimulus (Fig 1C) and phase modulation (Fig 1D) effects. Our approach can thus accurately recover the statistical relationships between firing rate and various external factors.

The model in Eq (1) is a “full” model including stimulus, auto-history, and an oscillatory factor. Importantly, we can remove selected factors from the full model (e.g., the LFP phase modulation) and still fit the spike trains using the same procedure. Indeed, in the following results, we also fit a simplified model lacking the oscillatory factor,

$$\begin{array}{c} \log\lambda \left(t|H_{t},X_{t}\right)=\log\lambda_{1}\left(t\right)+\log\lambda_{2}\left(t-t^{*}\right). \end{array}$$ (2)

### Estimation of LFP Phase Modulation

Many researchers have reported that firing rate is modulated by the phase of specific network-wide oscillations in different brain areas, such as monkey V1 [20], rat hippocampus [21], rat prefrontal cortex [22], mouse olfactory bulb [23], human pedunculopontine nucleus [24], lamprey reticulospinal neuron [25], and so on. Almost all of these results [20–22, 24, 25] used spike phase histograms to show how firing rate is modulated by the oscillation. The significance of phase locking can be evaluated using Rayleigh’s Z statistic [22]. The model in Eq (1) offers an alternative method of computing LFP phase modulation.

We simulated *N* spike trains and estimated LFP phase modulation using two different methods: 1) the classical spike phase histogram, and 2) by fitting *λ*_3_(*ϕ*) with the point process regression of model (1). The true LFP phase modulation function is defined as *λ*_3_(*ϕ*). The discrepancy between the estimated ${\hat{\lambda }}_{3}(\phi )$ and *λ*_3_(*ϕ*) is measured by the integrated squared error (ISE):

$$\begin{array}{c} \text{ISE}=\int_{-\pi }^{\pi }{\left[{\hat{\lambda }}_{3}\left(\phi \right)-\lambda_{3}\left(\phi \right)\right]}^{2}d\phi \end{array}$$

Using each method, we can derive point-by-point standard errors and 95% confidence bands for the LFP phase modulation. The mean integrated squared error (MISE) is then defined as:

$$\begin{array}{cc} \text{MISE} & =\frac{1}{n}\sum_{i=1}^{n}{\text{ISE}}_{i}\\ & =\frac{1}{n}\sum_{i=1}^{n}\int_{-\pi }^{\pi }{\left[{\hat{\lambda }}_{3}^{i}\left(\phi \right)-\lambda_{3}\left(\phi \right)\right]}^{2}d\phi , \end{array}$$

where *n* is the total number of data sets and *i* is the index of *i*th data set. ${\hat{\lambda }}_{3}^{i}(\phi )$ is computed given *N* repeated trials of spike train in *i*th data set. We can decompose MISE in terms of the sample mean ${\bar{\lambda }}_{3}(\phi )$ in the form of:

$$\begin{array}{c} \int_{-\pi }^{\pi }\{\frac{1}{n}\sum_{i=1}^{n}{\left[{\hat{\lambda }}_{3}^{i}\left(\phi \right)-{\bar{\lambda }}_{3}\left(\phi \right)\right]}^{2}+{\left[{\bar{\lambda }}_{3}\left(\phi \right)-\lambda_{3}\left(\phi \right)\right]}^{2}\}d\phi \end{array}$$

which provides an estimator of variance plus bias squared.

**Fig 2:**
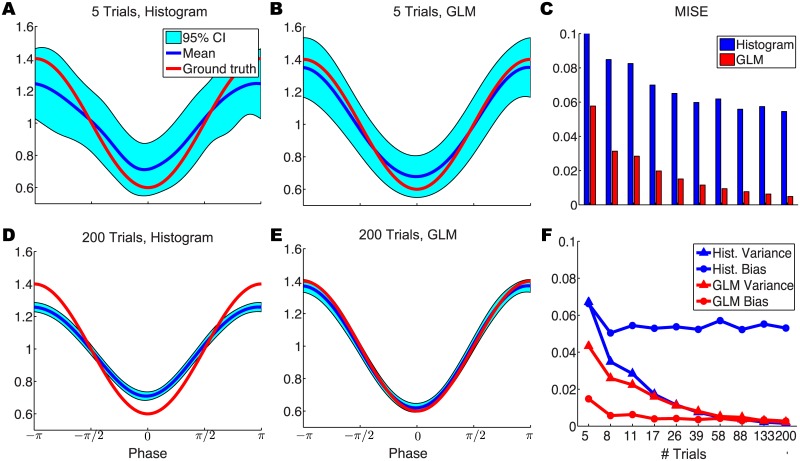
Estimation of LFP phase modulation by spike phase histogram and GLM methods. (A,B,D,E) Point process regression using the GLM (B,E) yields estimates of the LFP phase modulation with comparable variance but substantially lower bias than estimates made using the spike phase histogram method (A,D). (C) Comparison of the MISE between the estimated and true LFP phase modulation using the spike phase histogram and GLM methods, across different sample sizes. (F) Comparison of the variance and bias in the LFP phase modulation estimated by the two methods.

The histogram method is highly dependent on the bin size for smoothing. We picked the optimal bin size that minimizes the MISE. Fig 2C illustrates how the MISEs of the two methods are dependent on number of trials *N*. Both methods achieve smaller MISEs when more data are used, but the spike phase histogram method consistently exhibits a much larger MISE than the GLM method. Indeed, the spike phase histogram MISE reaches an asymptote for high *N* that is much larger than the MISE of the GLM method. In Fig 2F we show the variance and bias separately for the two methods. These results show that the spike phase histogram method retains a large bias, explaining the MISE asymptote in Fig 2C. The LFP phase modulation estimated by the spike phase histogram method additionally exhibits significantly larger variance than the GLM method for small sample sizes (< 17 trials).

Two additional comments can be made about the results shown above (Fig 2). First, when few trials or samples are available, only the GLM method can provide an accurate estimation of the LFP phase modulation of a neuron’s firing. Second, for moderately large samples, the error in the estimation of the LFP phase modulation by the spike phase histogram method arises primarily from estimation bias. We can explain this second point by considering the definitions of the two methods. The term *λ*_3_(*ϕ*) describes how an oscillation changes the firing rate and is independent of other factors (stimulus, auto-history, etc.). In contrast, the spike phase histogram method provides the distribution of phases when a spike occurs, denoted as *P*_*data*_(*ϕ*). Since the generation of a spike train is influenced by factors other than the oscillation, especially for a non-Poisson process, *P*_*data*_(*ϕ*) is conceptually different than *λ*_3_(*ϕ*). Below, we explore this conceptual difference further.

#### Bias in the estimation of the LFP phase modulation using the spike phase histogram method

To simplify our GLM, let us assume that the stimulus effect is a constant *λ*_1_(*t*) = *C* and *f* is the frequency of the oscillation. Now the firing probability model is:

$$\begin{array}{c} \lambda \left(t|H_{t},\phi_{t}\right)=C\cdot \lambda_{2}\left(t-t^{*}\right)\cdot \lambda_{3}\left(\phi_{t}\right). \end{array}$$

Suppose we observed a spike train {*u*_*k*_}. The spike phase histogram method provides an estimate of the distribution of {*ϕ*_*u*_*k*__},

$$\begin{array}{cc} P_{data}\left(\phi \right) & =P_{data}\left(\phi_{u_{k}}=\phi \right)\\ & =\int P_{data}\left(\phi_{u_{k-1}}=\phi_{0}\right)\cdot P_{data}\left(\phi_{u_{k}}=\phi |\phi_{u_{k-1}}=\phi_{0}\right)d\phi_{0}\\ & =\int P_{data}\left(\phi_{0}\right)\cdot P_{data}\left(\phi_{u_{k}}=\phi |\phi_{u_{k-1}}=\phi_{0}\right)d\phi_{0} \end{array}$$ (3)

where *P*_*data*_(*ϕ*_*u*_*k*__ = *ϕ*∣*ϕ*_*u*_*k* − 1__ = *ϕ*_0_) is the conditional probability of *ϕ*_*u*_*k*__ = *ϕ* given the phase of its previous spike is *ϕ*_0_. This conditional probability can be computed using the distribution of the waiting times [19] (page 602),

$$\begin{array}{c} f\left(t|H_{t},\phi_{t}\right)=\lambda \left(t|H_{t},\phi_{t}\right)\text{exp}\{-\int_{u_{k-1}}^{t}\lambda \left(u|H_{u},\phi_{u}\right)du\}. \end{array}$$

If we have a spike at phase *ϕ*, while its previous spike is at phase *ϕ*_0_, then the waiting time should be within the set $W=\{\Delta u:\Delta u=\frac{\phi -\phi_{0}}{w}+\frac{k}{f},\Delta u>0,k=0,1,2,\cdots ,w=2\pi f\}$. Thus

$$\begin{array}{cc} P_{data}\left(\phi_{u_{k}}=\phi |\phi_{u_{k-1}}=\phi_{0}\right) & =\frac{1}{w}\sum_{\phi_{t}=\phi ,t>u_{k-1}}f\left(t|H_{t},\phi_{t}\right)\\ & =\frac{1}{w}\sum_{\Delta u\in W}f\left(t=u_{k-1}+\Delta u|H_{t},\phi_{t}\right) \end{array}$$ (4)

Eq (3) shows that *P*_*data*_(*ϕ*) is an eigenfunction of *P*_*data*_(*ϕ*_*u*_*k*__ = *ϕ*∣*ϕ*_*u*_*k* − 1__ = *ϕ*_*u*_*k* − 1__). This is very hard to compute analytically, but we can get its numerical solution instead. When we discretize *ϕ* and write *P*_*data*_(*ϕ*) as a vector *P*, Eq (3) can be rewritten as

$$\begin{array}{c} P=A\cdot P \end{array}$$

where *P* ∈ ℝ^*m* × 1^, *m* is the number of bins to discretize *ϕ* ∈ [−*π*,*π*), and *A*∈ℝ^*m* × *m*^ is the transition probability

$$\begin{array}{c} A_{ij}=P_{data}\left(\phi_{u_{k}}=P_{i}|\phi_{u_{k-1}}=P_{j}\right). \end{array}$$

**Fig 3:**
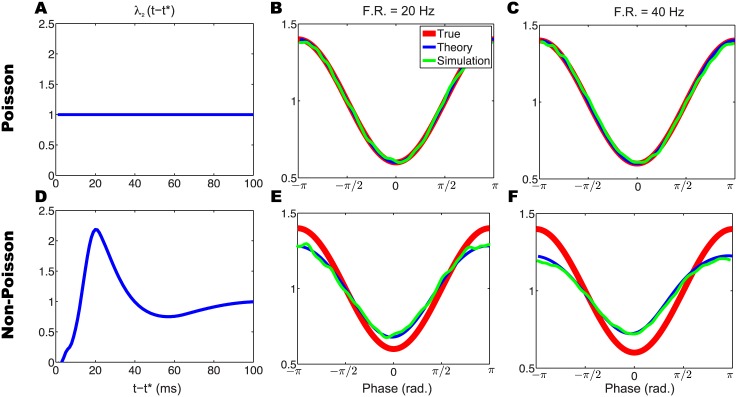
LFP phase modulation estimated by the spike phase histogram method is inherently biased for non-Poisson firing. (A,D) Auto-history effects for Poisson (A) and non-Poisson (D) firing. (B,C,E,F) Theoretical and simulated estimations of the LFP phase modulation for Poisson (B,C) and non-Poisson (E,F) firing at low (B,E) and high (C,F) mean firing rates. Note that, for non-Poisson firing, the spike phase histogram estimation of the LFP phase modulation introduces a firing rate-dependent bias.

Thus *P* is the eigenvector of *A* and its corresponding eigenvalue is 1. Numerical tools were used to compute *P*, which is the discrete version of *P*_*data*_(*ϕ*). In this way we can theoretically determine the LFP phase modulation given by the spike phase histogram. This theoretical prediction accurately predicts the LFP phase modulation estimated from simulated spike trains using the spike phase histogram method (Fig 3B, 3C, 3E and 3F).

Using this theoretical prediction, we examined how bias emerges in spike phase histogram estimates of the LFP modulation. We considered Poisson and non-Poisson firing across different mean firing rates. When a neuron’s firing approximates a Poisson process (i.e., *λ*_2_(*t*) = 1) (Fig 3A), the results of the spike phase histogram match *λ*_3_(*ϕ*) independent of the mean firing rate *C* (Fig 3B and 3C). Indeed, it can be shown that $P_{data}(\phi )=\frac{\lambda_{3}(\phi )}{2\pi }$ is a solution to Eq (3). Specifically, Eq (4) inserted into Eq (3) with some basic substitutions yields:

$$\begin{array}{c} \begin{array}{cc} 2\pi P_{data}\left(\phi \right)= & \lambda_{3}\left(\phi \right)\int_{0}^{\infty }C\cdot \lambda_{2}\left(t\right)\cdot 2\pi P_{data}\left(\phi -2\pi ft\right)\\ & \cdot \exp\{-\int_{0}^{t}C\cdot \lambda_{2}\left(t-u\right)\cdot \lambda_{3}\left(\phi -2\pi fu\right)du\}dt \end{array} \end{array}$$ (5)

When *λ*_2_(*t*) = 1, we replace *P*_*data*_(*ϕ*) with $\frac{\lambda_{3}(\phi )}{2\pi }$ in Eq (5). Then the integrand in the right side is $C\cdot \lambda_{3}(\phi -2\pi ft)\cdot \text{exp}\left\{-\int_{0}^{t}C\cdot \lambda_{3}(\phi -2\pi fu)du\right\}$, which is a probability density function and hence has the integral of 1, making the right side *λ*_3_(*ϕ*). Thus, *λ*_3_(*ϕ*) is one solution of Eq (3). These results show that the spike phase histogram estimate of the LFP phase modulation is accurate for Poisson firing. In contrast, for non-Poisson firing (in which the neuron’s firing rate is influenced by its firing history) *λ*_2_(*t*) is no longer a constant (Fig 3D). As a result, estimates of the LFP phase modulation curve diverge from *λ*_3_(*ϕ*) in a firing rate-dependent manner (Fig 3E and 3F). Thus, the GLM method for estimating a neuron’s LFP phase modulation is more accurate than the spike phase histogram method for smaller sample sizes (Fig 2) and for non-Poisson firing (Fig 3).

### Comparison with Spike Field Coherence

Spike field coherence (SFC) is commonly used to report interactions between spikes and specific oscillations in LFP. Lepage et al. [17, 26] showed that SFC is dependent on the expected rate of spiking, and they proposed to use intensity field coherence, which is a rate-independent measure, for inference of spike field synchrony. They also used GLMs to estimate spike field association [17]. In their work, they assumed that the LFP phase modulation is a sinusoidal function with period of 2*π*, which might not be accurate enough in some cases [22, 23]. In our model, to approximate this periodic function we use circular splines [18], which remain easy to fit while being more flexible than a sinusoidal function.

**Fig 4:**
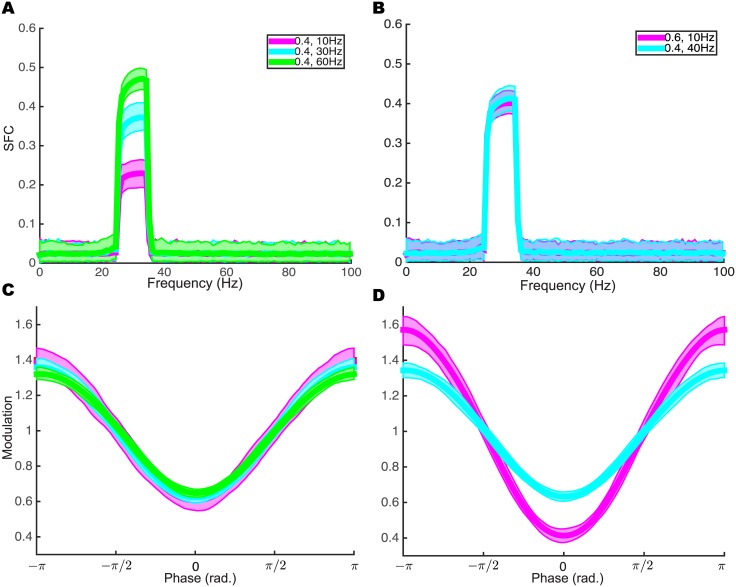
LFP phase modulation estimated by the GLM method does not depend on firing rate. (A,C), In three simulations, we keep *λ*_3_(*ϕ*) = 1 + 0.4cos(*ϕ*+*π*) while varying mean firing rates. The SFC method (A) reports three distinct results, while the GLM method (**C**) showed that the LFP phase modulations are the same. (B, D), Different combinations of firing rate and LFP phase modulation *λ*_3_(*ϕ*) = 1 + *a* ⋅ cos(*ϕ* + *π*) can yield the same SFC (B), while the GLM method can distinguish the differences in LFP phase modulation (D). For each parameter set (*a*,firing rate), we had 200 runs. The shaded area is the 95% confidence band.

Here, we provide two examples showing that when estimating spike field relationships, the SFC can be misleading. First, we simulated spike trains with three different mean firing rates, then computed SFCs with GNU software Chronux [27]. Fig 4A shows that the three SFCs are different even though they were generated by the same *λ*_3_(*ϕ*) = 1 + 0.4cos(*ϕ* + *π*). On the other hand, when we use our model to fit the LFP phase modulation functions, Fig 4C shows that there is no difference in phase modulation strength in these three cases. Second, we show that two neurons exhibiting different LFP phase modulations can have the same SFC (Fig 4B) because they have different firing rates. Again, we can use our model to distinguish these two conditions by their respective LFP phase modulation curves (Fig 4D).

### Synchrony and Oscillatory Phase

We now use point process regression of our GLMs Eqs (1) and (2) to analyze the contribution of network-wide oscillations to the synchronous spiking of two neurons. We first present numerical simulation results where ground truth is known and then apply the same technique to experimental neural recordings.

#### Simulation results

**Fig 5:**
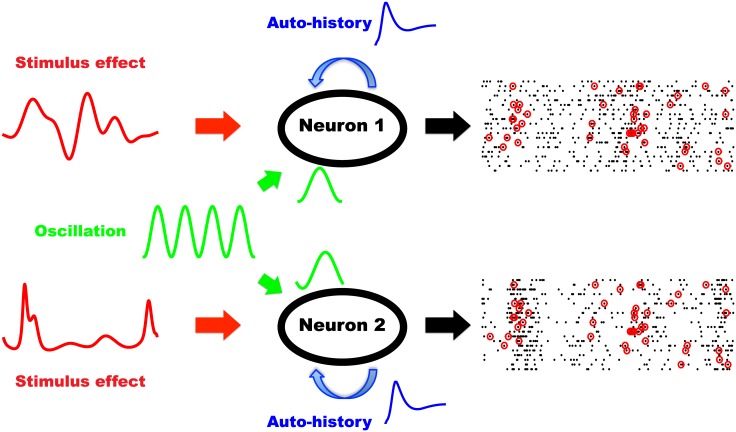
Schematic illustration of the contribution of a network-wide oscillation to synchronous spiking between two neurons. The firing probability of each neuron is influenced by three factors: stimulus, auto-history and an oscillatory drive. The oscillatory drive is shared by the two neurons, but each neuron exhibits a unique phase modulation curve. Spike trains of the two neurons are observed and synchronized spikes are counted (red circles).

In the Introduction, we described how GLMs can be used to assess the role of some potentially relevant factors in modulating spike synchrony. We designed a scenario in which we tested the contribution of a network-wide oscillation (i.e., an oscillatory LFP) to the number of synchronous spikes observed. This scenario is illustrated schematically in Fig 5 for two neurons. The stimulus effects (i.e., the tuning) of the two neurons are different, and both neurons’ spiking activities are influenced by their own recent spike histories. Critically, these two neurons also receive a common oscillatory signal with phase Φ_*t*_ that modulates their firing rate, but their individual phase modulation curves are shifted (i.e., they have different preferred phases Φ_*pref*_). Because the preferred phase modulates the average timing of each spike in one oscillatory cycle (in this example, ∼ 10 ms), differences in preferred phase lead to a relative shift in spike timing between the two neurons. The larger this shift, the less synchronized are their spikes. As a result, the observed number of synchronized spikes is dependent on the difference of preferred phase ΔΦ_*pref*_.

This simple scenario was used to demonstrate the effectiveness of the procedure, in principle, and to investigate its statistical power. The assumption that two neurons have different phase modulation curves has been reported both experimentally [20, 28] and theoretically [29]. Jia et al. [20] have shown that neurons in area V1 have various preferred phases and the distribution of the preferred phase can change in response to different stimuli. Richardson [29] computed analytically the modulation of the oscillatory signal for an exponential integrated-and-fire neuron. He showed that the modulation is influenced by biophysical properties of the neuron. He also showed that there is a phase lag between the peak firing rate and the peak of the oscillatory signal, which corresponds to the preferred phase in *λ*_3_(*ϕ*), and this phase lag is dependent on properties of the neuron. Usually the oscillations near two neurons in a small area are very similar; thus the assumption that two neurons receive a common modulation is reasonable. For two neurons located far apart (e.g., two brain areas), this assumption should be useful as long as the two oscillations are coherent. This more general case is relevant to hypotheses about mechanisms of neural communication [4, 20].

**Fig 6:**
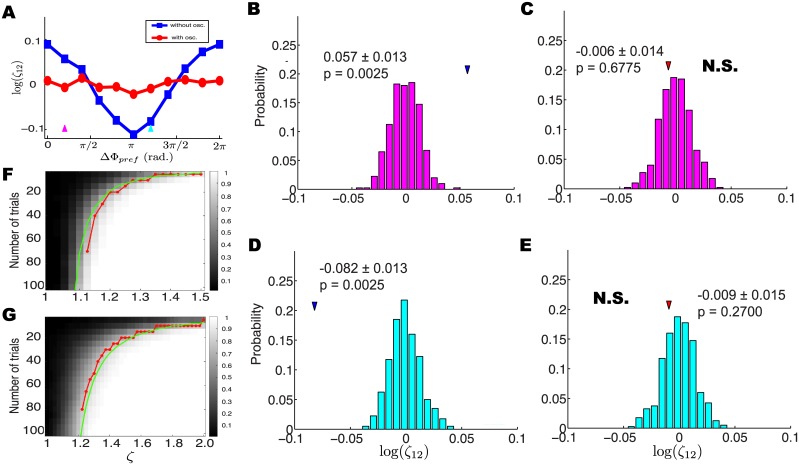
Network-wide oscillations can enhance or suppress the predicted levels of spike synchrony. (A) Dependence of $\text{log}\;{\hat{\zeta }}_{12}$ on the difference in preferred phases between two neurons, as computed using models with and without an oscillatory factor. Purple and cyan arrows indicate two different Δ(Φ_*pref*_)s. (B) Bootstrap-generated distribution of $\text{log}\;{\hat{\zeta }}_{12}$ values under the null hypothesis of log*ζ*_12_ = 0. Arrowhead shows the value of $\text{log}\;{\hat{\zeta }}_{12}$ computed by the simplified model. Thus, a significantly larger number of synchronous spikes is observed than predicted by the model lacking an oscillatory factor ($\text{log}\;({\hat{\zeta }}_{12})=0.057\pm 0.013$, *p* value = 0.0025). (C) Including an oscillatory factor in the model yields an accurate prediction of the observed number of synchronous spikes ($\text{log}({\hat{\zeta }}_{12})=-0.006\pm 0.014$, *p* value = 0.6775). (D, E) Same as (B,C) for different preferred phases that lead to significantly lower synchrony than predicted when an oscillatory factor is not included in the model (D: $\text{log}({\hat{\zeta }}_{12})=-0.082\pm 0.013$, *p* value = 0.0025; E: $\text{log}({\hat{\zeta }}_{12})=-0.009\pm 0.015$, *p* value = 0.2700). (F) Dependence of the power on number of trials and *ζ*. The mean firing rate is 25 Hz. The red and green lines indicate choices of *ζ* and *N* for which the power equals 0.8, based on simulation and theory respectively. (G) Same as (F), but the mean firing rate is 10 Hz.

To demonstrate directly the relationship between an oscillatory LFP and spike synchrony, we simulated spike trains from two neurons, then fitted models (1) and (2). For each model we used the estimator

$$\begin{array}{c} {\hat{\zeta }}_{12}=\frac{\text{Observed}\;\text{number}\;\text{of}\;\text{synchronized}\;\text{spikes}}{\text{Predicted}\;\text{number}\;\text{of}\;\text{synchronized}\;\text{spikes}} \end{array}$$

of its theoretical counterpart *ζ*_12_ defined in [16]. Under conditional independence, we have log *ζ*_12_ = 0, while conditional dependence yields either excess synchrony (log *ζ*_12_ > 0) or suppressed synchrony (log *ζ*_12_ < 0). We tested *H*_0_ : log *ζ*_12_ = 0 using a parametric bootstrap (see Materials and Methods). Results are shown in Fig 6. Using model (2) (i.e., without the oscillatory factor) we found that $\text{log}\;{\hat{\zeta }}_{12}$ is dependent on ΔΦ_*pref*_. This is because the relative phase preference of the two neurons changed the observed number of synchronized spikes, while the predicted number is almost the same when the contribution of the oscillatory LFP is disregarded. In contrast, when we included the oscillatory factor according to Eq (1), we found $\text{log}\;{\hat{\zeta }}_{12}$ to be close to 0 and independent of ΔΦ_*pref*_. Thus, including the oscillatory factor in our model removes the apparent conditional dependence of the predicted spike synchrony on the relative phase preference of the two neurons, and we can conclude that spike synchrony is associated with the oscillatory phase.

We picked two different values of ΔΦ_*pref*_ (purple and cyan arrows in the Fig 6A) to demonstrate the described hypothesis test. In the first example, we obtained evidence against the null hypothesis of log *ζ*_12_ = 0 using the simplified model (Fig 6B). That is, there is evidence that the two neurons are not conditionally independent given only the stimulus effects and spike history effects: they exhibit significant levels of excess spike synchrony. Fig 6C shows that including the oscillatory factor accounts for this excess synchrony. In other words, consideration of the oscillatory LFP can explain the higher than expected levels of spike synchrony predicted by the stimulus and spike history effects alone. In turn, lower than expected levels of spike synchrony predicted by the stimulus and spike history effects alone can be explained by consideration of the oscillatory LFP in the second example, in which the oscillatory LFP suppresses spike synchrony (Fig 6D and 6E).

We also investigated the amount of data needed to reliably detect excess synchrony by generating spike trains with varying numbers of trials, varying values of *ζ*, and two levels of firing rate, and then computing the probability of rejecting the null hypothesis (i.e., the statistical power). Fig 6F displays the power when we used the same simulation parameters (apart from *ζ* and number of trials) as in Fig 6A–6E. A standard target for power in the statistics literature is 0.8, and we have indicated this level of power with a red line in Fig 6F. Thus, to attain this high level of power when *ζ* = 1.125 we need 70 trials, but when *ζ* = 1.4 we need only 5 trials. This number is also highly dependent on the mean firing rate. When we change the firing rate from 25 Hz to 10 Hz, we need much more data to detect excess synchrony (Fig 6G). The simulation procedure is computationally slow, but a fast approximation is given by

$$\begin{array}{c} N=\lceil \frac{1}{T\lambda_{1}\lambda_{2}\delta }{\left(\frac{\Phi^{-1}\left(0.95\right)-\Phi^{-1}\left(0.2\right)/\sqrt{\zeta }}{\log\;\zeta }\right)}^{2}\rceil \end{array}$$

where *T* is the length of one trial, *λ*_1_ and *λ*_2_ are mean firing rates of two neurons, and *δ* is the bin size for detecting synchronized spikes. The approximate power from this formula is given by the green curves in Fig 6F and 6G. The formula is derived in **Materials and Methods**.

### Applications to Experimental Neural Recordings

To further demonstrate the value of our approach, we next examined the relationship between an oscillatory signal and spike synchrony in experimental neural recordings from two distinct preparations: hippocampal CA1 pyramidal cells recorded *in vitro* and V4 neurons recorded *in vivo*.

#### Hippocampal CA1 pyramidal cells

**Fig 7:**
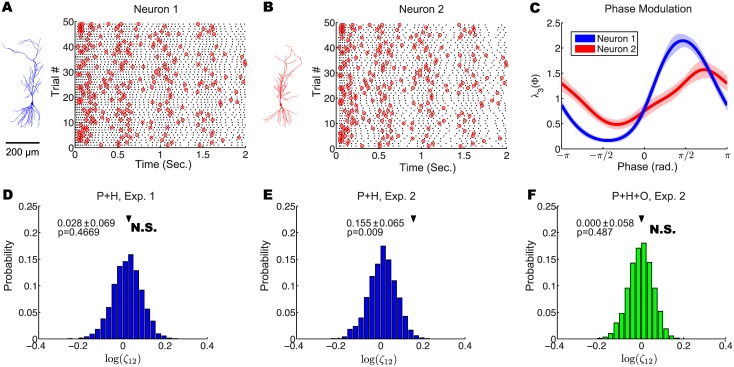
Shared oscillations contribute to spike synchrony between hippocampal CA1 pyramidal cells *in vitro*. (A, B) Reconstructed morphologies (left) and raster plots of spike trains (right) evoked in two CA1 pyramidal cells by an arbitrary stimulus waveform with a shared oscillatory signal (“Exp. 2”). Red circles show synchronized spikes between the two neurons. (C) Estimated phase modulation of the two recorded neurons in response to a shared oscillatory signal simulating a network-wide oscillation. (D) In the absence of a shared oscillatory signal, the simplified model (stimulus, or PSTH effects [P] + spike or auto-history effects [H]) lacking an oscillatory factor accurately predicts the observed number of synchronous spikes between the two neurons. (E,F) In the presence of a shared oscillatory signal, the simplified model (P + H) fails to explain the observed number of synchronous spikes (E) while the full model (stimulus, or PSTH effects [P] + spike or auto-history effects [H] + an oscillatory factor [O]) containing an oscillatory factor accurately predicts the observed number of synchronous spikes (F).

We first designed an experiment to resemble the scenario proposed in Fig 5 using whole-cell patch clamp recordings in a controllable acute slice preparation. In this experiment, we recorded the spiking response of, and spike synchrony between, two CA1 pyramidal cells (Fig 7A and 7B) in response to an arbitrary stimulus with and without a shared oscillatory signal. Critically, to directly test the relationship between the oscillatory signal and the resulting spike synchrony, we limited potential confounding influences on spike synchrony (e.g., common neuromodulatory influences, coupling between two neurons) by recording these neurons sequentially in two separate slices. Each neuron was injected with 100 trials of a 2 s-long 150 pA step current overlaid with a slow sinusoidal current (2 Hz frequency, 25 pA amplitude) and white noise (*σ* = 10 pA) to evoke physiological spike trains with low trial-to-trial reliability. The slow 2 Hz component was the same for all trials, and it generated visible time-varying fluctuations that are visible in the raster plots in Fig 7A and 7B, which led to a time-varying PSTH that was captured by the *λ*_1_(*t*) term in Eq (1). On 50 random trials (“Exp. 2”), an additional sinusoidal current (40 Hz frequency, 15 pA amplitude) with random initial phase (but identical between the two neurons) was also injected to simulate a gamma frequency network-wide oscillation. The 40 Hz component was not consistent over trials due to varying initial phases (S1A Fig), and its effect is, therefore, not captured by *λ*_1_(*t*). Instead, this 40 Hz modulatory effect was captured through the term *λ*_3_(Φ_*t*_) in Eq (1).

Thus, in the 50 trials without the simulated network-wide oscillation (“Exp. 1”), each neuron fired according to the its own stimulus and auto-history effects, generating a certain level of largely spontaneous spike synchrony reflecting the neurons’ fluctuating stimulus-driven firing rates. Using our simplified model (Eq (2)), we fit the spike trains from “Exp. 1” and predicted the number of synchronous spikes. As expected, the observed and predicted number of synchronous spikes closely matched (Fig 7D), consistent with the two neurons being conditionally independent given the arbitrary stimulus waveform and their own recent spiking histories. That is, no other factors were necessary to explain the observed number of synchronous spikes. However, using our simplified model to fit the spike trains from “Exp. 2” (Fig 7A and 7B), we observed a significantly greater number of synchronous spikes than could be explained by the stimulus and the neurons’ spike histories alone (Fig 7E). This conditional dependence between the two neurons arose because the firing of the two neurons was modulated by the simulated network-wide oscillation (Fig 7C). Indeed, using our full model (Eq (1)) to fit the spike trains from “Exp. 2” (S1B, S1C and S1D Fig), the number of synchronous spikes observed closely matched the number of synchronous spikes predicted (Fig 7F).

This experiment demonstrates that when two experimentally recorded neurons are not modulated by a shared oscillatory signal, then the simplified model (Eq (2)) can account for the observed number of synchronous spikes. However, when two neurons are modulated by a shared oscillatory signal (such as an oscillatory LFP, reflecting a network-wide oscillation), then a model including this oscillatory factor (Eq (1)) is necessary to account for the observed number of synchronous spikes. In contrast with our simulation above, the firing of these CA1 neurons is not described by the GLM in Eq (1) exactly. This model mismatch did not restrict the application of our method.

#### V4 neurons

**Fig 8:**
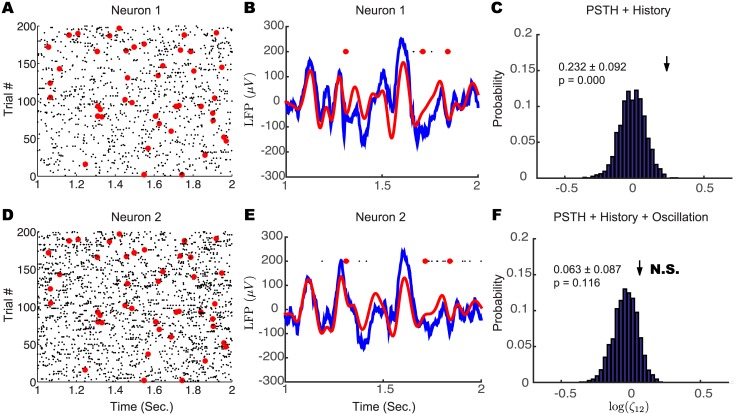
Shared oscillations contribute to spike synchrony between V4 neurons *in vivo*. (A,D) Raster plot of spike trains from two neurons recorded simultaneously. Red circles show synchronized spikes between the two neurons. (B,E) Raw (blue) and 4–25 Hz filtered (red) surrounding LFP related with each neuron for a single trial. (C,F) The simplified model failed to explain the observed number of synchronous spikes (C), while the full model containing an oscillatory factor fully accounts for the observed number of synchronous spikes.

In this experiment, spike trains from a pair of neural units in V4 were simultaneously recorded (Fig 8A and 8D) with a multi-electrode array during a fixation task in which spontaneous activity was measured. These data have been analyzed in another paper [28], which examined the relationship between individual neuron’s activity and large-scale network state. Here we wanted to test whether network-wide oscillations contribute to the excess pairwise synchrony. For each neuron, we defined its surrounding LFP as the average of LFPs recorded at its adjacent electrodes (see Materials and Methods). The spike-triggered average of the LFP for two neurons showed that the two neurons are phase locked to their surrounding field potential (S2C and S2D Fig and [28]). We also found that the LFP showed a prominent slow oscillation (Fig 8B and 8E). The LFP is thought to be the integrated effect of synaptic and spiking activity [30] near the recording sites. We filtered the LFP on each electrode within the 4–25 Hz band and extracted its phase to fit our full model (Eq (1)). Using the same procedure as in the case of the hippocampal CA1 pyramidal cells, we found that a significantly larger number of synchronous spikes were observed than could be explained by the simplified model (Fig 8C), while the full model fully explained the spike synchronization observed between the two neurons (Fig 8F). These results show that for these two neurons *in vivo*, spike synchronization is associated with the network-wide oscillation.

## Discussion

In this paper, we have shown how the GLM methods of [15–17, 26] may be combined in order to assess the potential contribution of network-wide oscillations to neural synchrony. The novel approach presented in this study complements existing alternatives [31–33] by: introducing models of single neuron firing based on stimulus-related fluctuations as well as a network-wide oscillatory signal; using those models to make predictions about spike synchronization; and quantifying departures from those predictions in the observed data. We demonstrated the advantages of this novel approach using both neural simulations and experimental neural recordings *in vitro* and *in vivo*.

In our analyses, we have utilized a repeated-trial structure, which allowed us to estimate the stimulus effects as a function of time, *λ*_1_(*t*). We note, however, that the same approach could be applied using a linear response filter [34–36] or analogous nonlinear methods. Previous work has shown the close relationship between GLM neurons and integrate-and-fire neurons [37–39]. We only considered one band of oscillation in simulation and experimental examples, but it is straightforward to extend this method to the case of multiple oscillations by including additional terms in the model of Eq (1). Sometimes the firing probability may be related to the amplitude of the oscillation *A*_*t*_, or the magnitude of an LFP *B*_*t*_ (cf. [17]). If so, we can change *f*_3_(Φ_*t*_) to *f*_3_(*A*_*t*_) or *f*_3_(*B*_*t*_). Overall, the key step of this method is to build an approximately correct GLM. The specific form of GLM depends on the data and we can check model performance using time rescaling [40]. We have also included a simulation to show that even when the model is mis-specified, and therefore less sensitive, it can detect spike-LFP relationships (S3 Fig). We have also defined spike synchrony to involve the firing of two neurons within a few milliseconds of each other (i.e., with zero lag on average). In other contexts, however, interest may focus on two neurons firing in procession with a consistent positive or negative lag of many milliseconds. Our approach could be easily applied to such lagged-synchrony cases as well.

In this paper, we consider only pairwise synchrony. By combining our approach with the procedure proposed by [16], we can also test the role of oscillations in three-way synchrony. Briefly, we fit all single neuron firing probabilities and then compute the pairwise synchrony coefficients ${\hat{\zeta }}_{ij}$; we can then use an iterative algorithm to estimate the three-way synchrony coefficient ${\hat{\zeta }}_{ijk}$, and to test the null hypothesis of two-way interactions, instead of three-way interaction. In principle the same steps may be followed for more than three neurons, but simulations in [16] show that very large data sets would be needed in order to demonstrate higher-order interactions convincingly in the absence of stronger assumptions about the nature of those interactions.

It has been argued that synchronous firing resulting from network-wide oscillations could provide an essential mechanism of network information flow, and further serve as a a marker distinguishing normal from diseased states (e.g., see [41–48]). On the other hand, there has been considerable debate on this subject (see [49] and references therein). We remain agnostic on this, and importantly, the value of our methods does not depend on the ultimate outcome of this debate. Instead, we view synchrony, more descriptively, as a feature of spike train data that needs to be explained. To this end, the framework that we have introduced here is useful for quantifying the extent to which oscillations, as a feature of neural activity, are associated with synchronous spiking among neurons. Armed with this method, future experiments can measure oscillations and synchrony in a statistical framework in which their contributions to cognitive and behavioral processes can be accurately quantified.

## Materials and Methods

### Point-Process Framework

In a continuous time interval (0,*T*], a neuron can fire a spike at any discrete time point *u*_*i*_. A series of spikes {*u*_*i*_} for 1 ≤ *i* ≤ *N* forms the spike train, where 0 ≤ *u*_1_ < ⋯ < *u*_*N*_ ≤ *T*. We take the spike train to be a point process, which is characterized by its conditional intensity function

$$\begin{array}{c} \lambda \left(t|H_{t},X_{t}\right)=\lim_{\Delta \to 0}\frac{P[N\left(t+\Delta \right)-N\left(t\right)=1|H_{t},X_{t}]}{\Delta } \end{array}$$ (6)

where *N*(*t*) is the total number of spikes prior to time *t*, *H*_*t*_ is neuron’s own spiking history prior to time *t*, and *X*_*t*_ includes all other relevant covariates. When Δ is small, *λ*(*t*∣*H*_*t*_,*X*_*t*_) ⋅ Δ approximates to the firing probability in the time interval (*t*, *t* + Δ). To determine how different factors contribute to firing rate we write *λ*(*t*∣*H*_*t*_,*X*_*t*_) as a function of (*H*_*t*_,*X*_*t*_)

$$\begin{array}{c} \lambda \left(t|H_{t},X_{t}\right)=f\left(H_{t},X_{t}\right). \end{array}$$ (7)

We can include different factors into this model and study their effects. Usually the stimulus *S*(*t*) is included when neurons show selectivity to stimuli. In this work, because we are interested in phase modulation by an oscillatory signal, the phase of the specific oscillation Φ(*t*) is also included.

### Generalized Linear Model and Maximum Likelihood Estimation

To take advantage of the generalized linear model (GLM) framework we divide T into K equally spaced bins, thus taking the bin width to be Δ = *T*/*K*. Δ is small enough that no more than one spike event in each bin, e.g. Δ = 1 ms. Therefore the probability of observing one spike in *k*th bin is

$$\begin{array}{c} p_{k}=\lambda \left(t_{k}|H_{t_{k}},X_{t_{k}}\right)\cdot \Delta ,\;k=1,2,\cdots ,K \end{array}$$ (8)

Using the vector *Y* ⊂ ℝ^*K* × 1^ to represent the spike train {*u*_*i*_}, *y*_*k*_ is the number of spikes in *k*th bin. Since we choose small bin width Δ, *y*_*k*_ is not bigger than 1, i.e. *y*_*k*_ ∈ {0,1} and, from the Poisson approximation to the binomial for small *p* we take the probability of observing *y*_*k*_ given *H*_*t*_*k*__ and *X*_*t*_*k*__ to be

$$\begin{array}{c} p\left(y_{k}|H_{t_{k}},X_{t_{k}}\right)=\frac{p_{k}^{y_{k}}}{y_{k}!}e^{-p_{k}} \end{array}$$ (9)

where *t*_*k*_ = *k*Δ. The loglikelihood function is

$$\begin{array}{c} L=\sum_{k=1}^{K}[y_{k}\cdot \log(p_{k})-p_{k}]=\sum_{k=1}^{K}[y_{k}\cdot \log(\lambda (t_{k}|H_{t_{k}},X_{t_{k}})\Delta )-\lambda \left(t_{k}|H_{t_{k}},X_{t_{k}}\right)\Delta ] \end{array}$$ (10)

and this is maximized to determine the MLE fit.

We assume that log[*λ*(*t*∣*H*_*t*_,*X*_*t*_)] can be written as a sum of specific functions of each covariate. Here we are studying three factors, stimulus, recent post-spike auto-history, and oscillatory phase, and we write

$$\log[\lambda (t|H_{t},X_{t})]=f_{1}\left(\text{stimulus}\right)+f_{2}\left(\text{auto-history}\right)+f_{3}\left(\text{oscillation}\right)$$ (11)

$$=f_{1}\left(S_{t}\right)+f_{2}\left(H_{t}\right)+f_{3}\left(\Phi_{t}\right).$$ (12)

Here, *S*(*t*) is a possibly time-varying stimulus and *f*_1_(stimulus) determines the trial-independent time-varying firing rate, i.e., the effect that is usually associated with the peri-stimulus time histogram (the PSTH), which may be estimated due to the repeated trial structure of the experiment. The recent post-spike auto-history effect is assumed here to be dominated by effects subsequent to the most recent spike t* prior to time *t*, as in [50], so we assume *f*_2_(auto-history) has the form *f*_2_(*t* − t*). The oscillatory term *f*_3_(oscillation) is defined as *f*_3_(Φ_*t*_), where Φ_*t*_ is the phase of specific oscillation. In summary, our spike train model has the form

$$\log(\lambda \left(t|H_{t},X_{t}\right))=f_{1}\left(t\right)+f_{2}\left(t-t^{*}\right)+f_{3}\left(\Phi_{t}\right)$$ (13)

$$=\log\lambda_{1}\left(t\right)+\log\lambda_{2}\left(t-t^{*}\right)+\log\lambda_{3}\left(\Phi_{t}\right)$$ (14)

and we will assume *f*_1_(⋅), *f*_2_(⋅) and *f*_3_(⋅) are smooth functions.

### Approximate Function with Spline Basis

To fit the smooth functions *f*_1_(⋅), *f*_2_(⋅) and *f*_3_(⋅) we use cubic splines of the form

$$\begin{array}{c} \log(\lambda (t|H_{t},X_{t}))=\sum_{i}a_{i}\left(t\right)\alpha_{i}+\sum_{j}b_{j}\left(t-t^{*}\right)\cdot \beta_{j}+\sum_{k}r_{k}\left(\Phi_{t}\right)\cdot \gamma_{k} \end{array}$$ (15)

where {*a*_*i*_(*t*)} is a B-spline basis set for *f*_1_(*t*) within the range *t* ∈ (0,*T*], $\left\{b_{j}(t-u_{t}^{*})\right\}$ is a B-spline basis set for *f*_2_(*t* − t*), and {*r*_*k*_(*ϕ*)} is circular spline basis set for *f*_3_(Φ_*t*_). Thus, we use maximum likelihood to fit the coefficients **Θ** = {*α*,*β*,*γ*}. We used open source software FDAfuns [51] to create each B-spline basis sets after manually selecting knots. For the circular spline we pick knots equally spaced in [−*π*,*π*]. Once we get all knots {*ϕ*_*i*_}, acquiring the related basis function is straightforward [18] using

$$\begin{array}{c} r_{k}\left(\phi \right)=\sum_{m=1}^{\infty }\frac{2}{{\left(2\pi m\right)}^{4}}\cos(2\pi m(\phi -\phi_{k})). \end{array}$$ (16)

In numerical implementations, we usually cut the summation from *m* = 1 to *m* = 4 because amplitude of each term decreases quickly.

### Maximum Likelihood via Iteratively Re-weighted Least Squares

Because *L* in Eq (10) is a concave function, we can use iteratively reweighted least squares (IRLS), as in typical GLM implementations. From Eqs (8), (10), and (15), we can rewrite loglikelihood in matrix form

$$\begin{array}{cc} L= & Y^{T}\cdot \log\mu -I_{1\times K}\cdot \mu \end{array}$$ (17)

$$\begin{array}{cc} \log\mu = & [A\cdot \alpha +B\cdot \beta +R\cdot \gamma ]\Delta \end{array}$$ (18)

Here we have three parameter sets to fit {*α*,*β*,*γ*}. If we fit all three parameter sets together, the dimension space of this GLM model is relative large. To make model fitting efficient, we prefer back-fitting, i.e., fitting each parameter set separately, and iterating cyclically. For example, when we fit the parameters {*α*}, we hold the parameters {*β*,*γ*} constant and rewrite Eq (18) as

$$\begin{array}{c} \log\mu =V\cdot \theta +\log\mu_{t}^{0} \end{array}$$ (19)

where *θ* ∈ {*α*,*β*,*γ*} and *V* is the corresponding covariate matrix. We fit {*α*,*β*,*γ*} in a sequence and then iterate the loop until convergence. We also must place identifiability restrictions on {*β*,*γ*} because both the auto-history and oscillatory effects modulate the spike trains and the parameters must be constrained to provide unique solutions. We use the constraints

$$\begin{array}{cc} \frac{\int_{0}^{T}\exp[\sum_{j}b_{j}\left(\tau \right)\cdot \beta_{j}]d\tau }{T}= & 1 \end{array}$$ (20)

$$\begin{array}{cc} \frac{\int_{-\pi }^{\pi }\exp[\sum_{k}r_{k}(\Phi )\gamma_{k}]d\Phi }{2\pi }= & 1. \end{array}$$ (21)

To avoid over-fitting of the model, we also add an *l*_2_ penalty into the objective function. Now the problem becomes minimizing objective function

$$\begin{array}{c} Q=-L+\frac{\lambda }{2}{\left|\Theta \right|}_{2}=-Y^{T}\cdot \left(V\cdot \Theta +\log\mu_{t}^{0}\right)+I_{1\times K}\cdot \exp\left(V\cdot \Theta +\log\mu_{t}^{0}\right)+\frac{\lambda }{2}\cdot \Theta^{T}\Theta . \end{array}$$ (22)

Because the objective function *Q* is convex, we can iteratively maximize Θ by following the updating rule

$$\begin{array}{c} \Theta^{i+1}=\Theta^{i}-H^{-1}\cdot \nabla Q \end{array}$$ (23)

where *H* is the Hessian of Q and ∇*Q* is the gradient of the function, which are obtained as

$$\begin{array}{cc} \nabla Q= & V^{T}[\exp(V\cdot \Theta +\log\mu_{t}^{0})-Y]+\lambda \Theta \end{array}$$ (24)

$$\begin{array}{cc} H= & V^{T}\cdot W\cdot V+\lambda \end{array}$$ (25)

where *W* is a diagonal matrix

$$\begin{array}{c} W_{i,j}=\{\begin{array}{cc} \exp{\left(V\cdot \Theta +\log\mu_{t}^{0}\right)}_{i} & \text{if}\;i=j\\ 0\; & \text{otherwise.} \end{array} \end{array}$$ (26)

The algorithm is summarized as **Algorithm 1**, shown below.

**Algorithm 1**: IRLS method for finding argmin_Θ_ *Q*(Θ)

**Data**: $Y,V,\Theta_{0},\text{log}\mu_{t}^{0},\lambda$

**Result**: Θ* = argmin_Θ_ *Q*(Θ)

**begin**

*Q*_1_ ← *Q*(Θ_0_);

**repeat**

*Q*_0_ ← *Q*_1_;

$\nabla Q\leftarrow V^{T}\left[\text{exp}(V\cdot \Theta_{0}+\text{log}\mu_{t}^{0})-Y\right]+\lambda \Theta_{0}$;

$W\leftarrow \text{diag}\left\{\text{exp}(V\cdot \Theta_{0}+\text{log}\mu_{t}^{0})\right\}$;

*H* ← *V* ^*T*^ ⋅ *W* ⋅ *V*+*λ*;

   Θ_1_ ← Θ_0_ − *H* ^−1^ ⋅ ∇*Q*;

*Q*_1_ ← *Q*(Θ_1_);

   Θ_0_ ← Θ_1_;

**until** ∣*Q*_1_ − *Q*_0_∣ ≤ *δ*;

**return** Θ_1_;

**end**

### Spike Synchronization

For a pair of neurons labeled 1 and 2, we fit conditional firing rate for each of them to get ${\hat{\lambda }}_{1}(t|H_{t},X_{t})$ and ${\hat{\lambda }}_{2}(t|H_{t},X_{t})$. Then we can predict the number of synchronized spikes given temporal bins with Δ = 5*ms*, as in [15], using

$$\begin{array}{c} N_{pred}=\int {\hat{\lambda }}_{1}\left(t|H_{t},X_{t}\right)\cdot {\hat{\lambda }}_{2}\left(t|H_{t},X_{t}\right)dt. \end{array}$$ (27)

Given spike trains from these two neurons, we can also get the observed number of synchronized spikes *N*_*obs*_ by counting. If a pair of spikes from two neurons has an time interval less than 5 *ms*, then this pair is counted as a synchronized spike. Synchrony is measured by taking the ratio of these two numbers $\hat{\zeta }$ or $\text{log}\;\hat{\zeta }$

$$\begin{array}{c} \hat{\zeta }=\frac{N_{obs}}{N_{pred}}. \end{array}$$ (28)

When two neurons are conditional independent, Eq (27) can make relative good predictions and $\hat{\zeta }\approx 1$ (or $\text{log}\;\hat{\zeta }\approx 0$).

### Bootstrap Method

Once we have $\text{log}\;\hat{\zeta }$, we also need to determine its standard error and confidence interval. Furthermore, a *p* value is required to test the hypothesis $\text{log}\;\hat{\zeta }=0$. We use a parametric bootstrap method for these purposes, as in [16]. For example, given ${\hat{\lambda }}_{1}(t|H_{t},X_{t})$ and ${\hat{\lambda }}_{2}(t|H_{t},X_{t})$ we can obtain the *p* value as follows:

- For *i* in 1 : *G* do
  1. Simulate each of the two sets of spike trains, across the same number of trials as in the data, using the respective spike train models with ${\hat{\lambda }}_{1}$ and ${\hat{\lambda }}_{2}$.
  2. Compute log *ζ*_*i*_ from the spike trains generated in step 1.
- Compute the number of values {log *ζ*_*i*_} (out of a total of *G* such values) for which $|\left\{\text{log}\;\zeta_{i}\right\}|>|\text{log}\;\hat{\zeta }|$ and divide by *G*. This is the *p* value.

### Power Analysis

Statistical power is the probability of correctly rejecting the null hypothesis when it is false. We used the GLM model in Eq (1) to study power as a function of *ζ* and *N* (N being the number of trials). We simulated *N* trials of spike train data for each of two neurons, independently, using Eq (1) with intensity functions *λ* ^(1)^(*t*∣*H*_*t*_,*X*_*t*_) for the first neuron and *λ* ^(2)^(*t*∣*H*_*t*_,*X*_*t*_) for the second. The synchronous spikes in the resulting spike trains occur with probability corresponding to *ζ* = 1 (independence). In order to obtain sets of spike trains for other values of *ζ* we removed all the synchronous spikes from the *N* simulated spike trains and replaced them with synchronous spikes generated from an intensity function *ζ* ⋅ *λ*_1_(*t*∣*H*_*t*_,*X*_*t*_) ⋅ *λ*_2_(*t*∣*H*_*t*_,*X*_*t*_), i.e., for each time bin of width *δ*, synchronous spikes occurred with probability *ζ* ⋅ *λ*_1_(*t*∣*H*_*t*_,*X*_*t*_) ⋅ *λ*_2_(*t*∣*H*_*t*_,*X*_*t*_)*δ* ^2^. However, while this is the desired probability of synchronous spikes, it leaves the wrong marginal probability of spiking for each neuron. To adjust these we consider the spike trains made up of only the non-synchronous spikes, and we thin these with probabilities *p* ^(*j*)^(*t*) given by

$$\begin{array}{c} p^{\left(j\right)}\left(t\right)=\frac{\lambda^{\left(j\right)}(t|H_{t},X_{t})-\zeta \cdot \lambda^{\left(1\right)}(t|H_{t},X_{t})\cdot \lambda^{\left(2\right)}(t|H_{t},X_{t})\delta }{\lambda^{\left(j\right)}(t|H_{t},X_{t})-\lambda^{\left(1\right)}(t|H_{t},X_{t})\cdot \lambda^{\left(2\right)}(t|H_{t},X_{t})\delta } \end{array}$$

for *j* = 1,2. Note that when we multiply the numerator and denominator of this expression by *δ* we have the ratio of the desired probability of a non-synchronous spike to the probability of a non-synchronous spike under independence (the latter probability corresponding to the process we are thinning). After obtaining all *N* trials we then fitted the model to these simulated spike trains, found the estimate $\hat{\zeta }$, and applied the hypothesis test using the bootstrap method. This procedure was carried out for each *ζ* and *N* in our simulation.

Because the simulation is computationally time-consuming, for the benefit of any future efforts along these lines, we also derived a formula to approximate the number of trials needed to get 0.8 power. Suppose we have *N* trials, each trial is *T* seconds, the bin size for synchrony detection is *δ*. Denote the instantaneous firing rates for two neurons on trial *i* by $\lambda_{t,i}^{\left(1\right)}$ and $\lambda_{t,i}^{\left(2\right)}$. The number of synchronized spikes within the *t*th bin is $y_{t,i}^{\left(12\right)}$ and $y_{t,i}^{\left(12\right)}∼\text{Poisson}\left(\zeta \cdot \lambda_{t,i}^{\left(1\right)}\lambda_{t,i}^{\left(2\right)}\cdot \delta^{2}\right)$, where *ζ* is the synchrony coefficient. The total number of observed synchronized spikes given $\lambda_{t,i}^{\left(1\right)}$ and $\lambda_{t,i}^{\left(2\right)}$ is $N_{obs}|\lambda_{t,i}^{\left(1\right)},\lambda_{t,i}^{\left(2\right)}=\sum_{i=1}^{N}\sum_{t=1}^{T/\delta }y_{t,i}^{\left(12\right)}$. Then we compute $\hat{\zeta }$ conditioned on $\lambda_{t,i}^{\left(1\right)}$ and $\lambda_{t,i}^{\left(2\right)}$,

$$\begin{array}{c} \hat{\zeta }|\lambda_{t,i}^{\left(1\right)},\lambda_{t,i}^{\left(2\right)}=\frac{N_{obs}|\lambda_{t,i}^{\left(1\right)},\lambda_{t,i}^{\left(2\right)}}{N_{pred}}=\frac{\sum_{i=1}^{N}\sum_{t=1}^{T/\delta }y_{t,i}^{\left(12\right)}}{\sum_{i=1}^{N}\sum_{t=1}^{T/\delta }\lambda_{t,i}^{\left(1\right)}\lambda_{t,i}^{\left(2\right)}\cdot \delta^{2}}. \end{array}$$

Since $y_{t,i}^{\left(12\right)}∼\text{Poisson}\left(\zeta \cdot \lambda_{t,i}^{\left(1\right)}\cdot \lambda_{t,i}^{\left(2\right)}\delta^{2}\right)$, we can easily get

$$\begin{array}{cc} E[\hat{\zeta }|\lambda_{t,i}^{\left(1\right)},\lambda_{t,i}^{\left(2\right)}] & =\frac{E[\sum_{i=1}^{N}\sum_{t=1}^{T/\delta }y_{t,i}^{\left(12\right)}]}{\sum_{i=1}^{N}\sum_{t=1}^{T/\delta }\lambda_{t,i}^{\left(1\right)}\lambda_{t,i}^{\left(2\right)}\cdot \delta^{2}}=\frac{\sum_{i=1}^{N}\sum_{t=1}^{T/\delta }\zeta \cdot \lambda_{t,i}^{\left(1\right)}\lambda_{t,i}^{\left(2\right)}\delta^{2}}{\sum_{i=1}^{N}\sum_{t=1}^{T/\delta }\lambda_{t,i}^{\left(1\right)}\lambda_{t,i}^{\left(2\right)}\cdot \delta^{2}}=\zeta \\ Var(\hat{\zeta }|\lambda_{t,i}^{\left(1\right)},\lambda_{t,i}^{\left(2\right)}) & =\frac{Var(\sum_{i=1}^{N}\sum_{t=1}^{T/\delta }y_{t,i}^{\left(12\right)})}{{\left(\sum_{i=1}^{N}\sum_{t=1}^{T/\delta }\lambda_{t,i}^{\left(1\right)}\lambda_{t,i}^{\left(2\right)}\delta^{2}\right)}^{2}}=\frac{\sum_{i=1}^{N}\sum_{t=1}^{T/\delta }\zeta \cdot \lambda_{t,i}^{\left(1\right)}\lambda_{t,i}^{\left(2\right)}\delta^{2}}{{\left(\sum_{i=1}^{N}\sum_{t=1}^{T/\delta }\lambda_{t,i}^{\left(1\right)}\lambda_{t,i}^{\left(2\right)}\delta^{2}\right)}^{2}}\\ & =\frac{\zeta }{\sum_{i=1}^{N}\sum_{t=1}^{T/\delta }\lambda_{t,i}^{\left(1\right)}\lambda_{t,i}^{\left(2\right)}\delta^{2}}. \end{array}$$

Assuming $\lambda_{t,i}^{\left(1\right)}$ and $\lambda_{t,i}^{\left(2\right)}$ are independent, we have

$$\begin{array}{c} E[\sum_{i=1}^{N}\sum_{t=1}^{T/\delta }\lambda_{t,i}^{\left(1\right)}\lambda_{t,i}^{\left(2\right)}\delta^{2}]=\sum_{i=1}^{N}\sum_{t=1}^{T/\delta }E[\lambda_{t,i}^{\left(1\right)}]E[\lambda_{t,i}^{\left(2\right)}]\delta^{2}=NT\lambda_{1}\lambda_{2}\delta , \end{array}$$

where *λ*_1_ and *λ*_2_ are the mean firing rates of two neurons. Then we have

$$\begin{array}{cc} E[\hat{\zeta }] & =E[E[\hat{\zeta }|\lambda_{t,i}^{\left(1\right)},\lambda_{t,i}^{\left(2\right)}]]=\zeta \\ Var(\hat{\zeta }) & =Var(E[\hat{\zeta }|\lambda_{t,i}^{\left(1\right)},\lambda_{t,i}^{\left(2\right)}])+E[Var(\hat{\zeta }|\lambda_{t,i}^{\left(1\right)},\lambda_{t,i}^{\left(2\right)})]\\ & =\frac{\zeta }{NT\lambda_{1}\lambda_{2}\delta }+O(\frac{\zeta }{{\left(NT\lambda_{1}\lambda_{2}\delta \right)}^{3}})\\ E[\log\hat{\zeta }] & \approx \log\zeta -\frac{1}{\zeta^{2}}Var(\hat{\zeta })=\log\zeta +O(\frac{1}{NT\lambda_{1}\lambda_{2}\delta \zeta })\\ Var(\log\hat{\zeta }) & \approx \frac{1}{\zeta^{2}}Var\left(\hat{\zeta }\right)-\frac{1}{4\zeta^{2}}Var{\left(\hat{\zeta }\right)}^{2}=\frac{1}{\zeta }\frac{1}{NT\lambda_{1}\lambda_{2}\delta }+O(\frac{1}{{\left(NT\lambda_{1}\lambda_{2}\delta \right)}^{2}}). \end{array}$$

We next assume that the distribution of $\text{log}\;\hat{\zeta }$ is (approximately) normal, i.e., $\text{log}\;\hat{\zeta }\in N\left(\text{log}\;\zeta ,\frac{1}{\zeta }\frac{1}{NT\lambda_{1}\lambda_{2}\delta }\right)$, so that to get the power to equal 0.8 with type I error.05 we need

$$\begin{array}{cc} \Phi (\frac{x-\log\zeta }{\sqrt{\frac{1}{\zeta }\frac{1}{NT\lambda_{1}\lambda_{2}\delta }}})= & 0.2\\ \Phi (\frac{x-\log1}{\sqrt{\frac{1}{NT\lambda_{1}\lambda_{2}\delta }}})= & 0.95, \end{array}$$

where *x* is the threshold of rejecting null hypothesis log *ζ* = 0. We can then solve for *N* as the number of needed trials for detecting excess synchony:

$$\begin{array}{c} N=\lceil \frac{1}{T\lambda_{1}\lambda_{2}\delta }{\left(\frac{\Phi^{-1}\left(0.95\right)-\Phi^{-1}\left(0.2\right)/\sqrt{\zeta }}{\log\zeta }\right)}^{2}\rceil . \end{array}$$

### Experiment

#### Acute slice electrophysiology

Experiments were completed in compliance with the guidelines established by the Institutional Animal Care and Use Committee of Carnegie Mellon University. Whole-cell patch clamp recordings of hippocampal CA1 pyramidal cells were performed similar to previously described methods [52]. Briefly, a postnatal day 16 Thy1-YFP-G mouse [53] was anesthetized with isoflurane and decapitated into ice-cold oxygenated dissection solution containing (in *mM*): 125 NaCl, 25 glucose, 2.5 KCl, 25 NaHCO_3_, 1.25 NaH_2_PO_4_, 3 MgCl_2_ and 1 CaCl_2_. Brains were rapidly isolated and sagittal slices (310 *μ*m thick) containing the hippocampus were cut using a vibratome (5000 mz-2; Campden, Lafayette, IN, USA). Slices recovered for ∼ 30 min in ∼ 37C oxygenated Ringer solution that was identical to the dissection solution except for lower Mg^2+^ concentrations (1 mM MgCl_2_) and higher Ca^2+^ concentrations (2 mM CaCl_2_). Slices were then stored in room temperature oxygenated Ringer solution until recording. During recording, slices were continuously superfused with warmed oxygenated Ringer’s solution (temperature measured in bath: 32°C). CA1 pyramidal cells were identified by morphology and laminar position using infrared differential interference contrast microscopy. Whole-cell recordings were made using electrodes (final electrode resistance: 5–7 MΩ) filled with (in mM): 120 potassium gluconate, 2 KCl, 10 Hepes, 10 sodium phosphocreatine, 4 Mg-ATP, 0.3 Na_3_GTP, 0.2 EGTA, 0.25 Alexa Fluor 594 (Life Technologies, Carlsbad, CA, USA) and 0.2% Neurobiotin (Vector Labs, Burlingame, CA, USA). The liquid junction potential was 12–14 mV and was not corrected for. Pipette capacitance was carefully neutralized and series resistance was compensated using the MultiClamp Bridge Balance operation. Data were low-pass filtered at 4 kHz and digitized at 10 kHz using a MultiClamp 700A amplifier (Molecular Devices, Sunnyvale, CA, USA) and an ITC-18 acquisition board (Instrutech, Mineola, NY, USA) controlled by custom software written in Igor Pro (WaveMetrics, Lake Oswego, OR, USA). Cell morphology was reconstructed under a 100X oil-immersion objective and analyzed with Neurolucida (MicroBrightField, Inc., Williston, VT, USA).

#### V4 neurons

Experimental procedures were approved by the Institutional Animal Care and Use Committee of the University of Pittsburgh. A separate analysis of these data has been previously reported ([28, 54]).

**Subjects**: We implanted one, 100-electrode “Utah” array (Blackrock Microsystems) in right V4 in one adult male rhesus macaque (*Macaca mulatta*). The basic surgical procedures have been described previously [55], and were conducted in aseptic conditions under isoflurane anesthesia. In addition to the microelectrode arrays, the animal was implanted with a titanium head post to immobilize the head during experiments. We recorded neurons with receptive fields centered ∼ 4° from the fovea in the lower-left visual field.

***Behavioral task***: We trained the subject to maintain fixation on a 0.6° blue dot at the center of a flat-screen cathode ray tube monitor positioned 36 cm from its eyes. The background of the display was 50% gray. We measured the monitor luminance gamma functions using a photometer and linearized the relationship between input voltage and output luminance using lookup tables. The subject was trained to maintain fixation on the central dot for 2 seconds while no other visual stimulus was presented, at which time the fixation point was moved 11.6° in a random direction and the animal received a liquid reinforcement for making a saccade to the new location.

***Microelectrode array recordings***: Signals from the microelectrode arrays were band-pass filtered (0.3–7500 Hz), digitized at 30 kHz and amplified by a Grapevine system (Ripple). Signals crossing a threshold (periodically adjusted using a multiple of the root-mean-squared [RMS] noise for each channel) were stored for offline analysis. These waveform segments were sorted using an automated clustering algorithm [56] followed by manual refinement using custom MATLAB software [57] (available at http://www.smithlab.net/spikesort.html), taking into account the waveform shapes and interspike interval distributions. After sorting, we calculated the signal-to-noise (SNR) ratio of each candidate unit as the ratio of the average waveform amplitude to the standard deviation of the waveform noise [57]. Candidates with an SNR below 2.5 were discarded. Signals were also filtered from 0.3–250 Hz with a digital Butterworth filter and sampled at 1 kHz to provide LFPs.

***LFP preprocessing***: We assume that the oscillation modulating spiking activity is explicitly within the surrounding LFP. The naive way of selecting LFP is using the one recorded at the same electrode for each neuron. Since spike waveforms might contaminate the LFP spectrum [30, 58], we computed LFP related to each neuron as the average of LFPs recorded on its neighboring electrodes. Another way of avoiding spike bleed-through is to choose the LFP on any electrodes adjacent to the neuron. In S2A and S2B Fig, we show that LFPs selected by all three methods are very similar. We also computed the spike-triggered average (STA) field potential using these three different methods. Their shapes are almost the same (S2C ad S2D Fig). We then bandpass filtered the LFP using Chebyshev type II filter design with passband 4–25 Hz. After we got the filtered oscillatory signal (Fig 8B–8E), we applied the Hilbert transform to estimate the instantaneous phase for further model fitting [20].

## Supporting Information

S1 Fig GLM fitting of one CA1 neuron. (A) Input currents for two differnt trials. The slow 2 Hz components are the same, but the fast 40 Hz oscillatory signals are different due to the varying initial phases. Both input currents have white noise. (B) Effect of stimulus *λ*_1_(*t*). (C) Effect of auto-history *λ*_2_(*t* − t*). (D) Effect of phase modulation *λ*_3_(*ϕ*) from the oscillatory signal.
(EPS)

S2 Fig Spike triggered average of two V4 neuron. (A)(B) Three different ways of selecting the LFP for each neuron: LFP on the same electrode as the neuron detected (red), LFP on one of the neighboring electrodes (blue), averaged LFP on all neighboring electrodes (green); (C)(D) spike-triggered average for three different field potentials shown in (A)(B).
(EPS)

S3 Fig Explaining synchrony when firing rate is modulated by the amplitude of the oscillation. In this example, the firing rate is modulated by the magnitude of the oscillation *B*_*t*_ = *A*_*t*_ cos(Φ_*t*_), where the amplitude *A*_*t*_ is time varying and the modulation curve is 1 + *B*_*t*_ = 1 + *A*_*t*_cos(Φ_*t*_). We want to show that even though the phase-modulation assumption is violated, our method can still explain partly the role of oscillation in synchrony. (A) Amplitude and magnitude of the oscillatory signal; (B) Bootstrap-generated distribution of log *ζ*_12_ values under the null hypothesis of log *ζ*_12_. Arrowhead shows the value of log *ζ*_12_ predicted by the simplified model. A significantly larger number of synchronous spikes is observed than predicted by the model lacking an oscillatory factor. (C) Including an oscillatory factor in the model yields an accurate prediction of the observed number of synchronous spikes.
(EPS)

S1 Dataset Experimental dataset used in this paper. Two datasets used for Fig 7 and Fig 8 were included in S1_Dataset. They were named as CA1_data and V4_data respectively. Details of data format were described in README file of each dataset.
(ZIP)

## References

[1] Brette R. Computing with neural synchrony. PLoS Computational Biology. 2012;8 10.1371/journal.pcbi.1002561 22719243 PMC3375225

[2] Colgin LL, Denninger T, Fyhn M, Hafting T, Bonnevie T, Jensen O, et al Frequency of gamma oscillations routes flow of information in the hippocampus. Nature. 2009 11;462(7271):353–7. 10.1038/nature08573 19924214

[3] Engel AK, Fries P, Singer W. Dynamic predictions: oscillations and synchrony in top-down processing. Nature Reviews Neuroscience. 2001;2:704–716. 10.1038/35094565 11584308

[4] Fries P. A mechanism for cognitive dynamics: Neuronal communication through neuronal coherence. Trends in Cognitive Sciences. 2005;9:474–480. 10.1016/j.tics.2005.08.011 16150631

[5] Geman S. Invariance and selectivity in the ventral visual pathway. Journal of Physiology Paris. 2006;100:212–224. 10.1016/j.jphysparis.2007.01.001 17336506

[6] Niebur E, Hsiao SS, Johnson KO. Synchrony: A neuronal mechanism for attentional selection? Current Opinion in Neurobiology. 2002;12:190–194. 10.1016/S0959-4388(02)00310-0 12015236

[7] Salinas E, Sejnowski TJ. Correlated neuronal activity and the flow of neural information. Nature Reviews Neuroscience. 2001;2:539–550. 10.1038/35086012 11483997 PMC2868968

[8] Sejnowski TJ, Paulsen O. Network oscillations: emerging computational principles. The Journal of Neuroscience. 2006;26(6):1673–1676. 10.1523/JNEUROSCI.3737-05d.2006 16467514 PMC2915831

[9] Tiesinga P, Fellous JM, Sejnowski TJ. Regulation of spike timing in visual cortical circuits. Nature Reviews Neuroscience. 2008;9:97–107. 10.1038/nrn2315 18200026 PMC2868969

[10] Denker M, Roux S, Linden H, Diesmann M, Riehle A, Grun S. The Local Field Potential Reflects Surplus Spike Synchrony. Cerebral Cortex. 2011 4;21(12):2681–2695. 10.1093/cercor/bhr040 21508303 PMC3209854

[11] Friedrich RW, Habermann CJ, Laurent G. Multiplexing using synchrony in the zebrafish olfactory bulb. Nature Neuroscience. 2004;7(8):862–871. 10.1038/nn1292 15273692

[12] Gregoriou GG, Gotts SJ, Zhou H, Desimone R. High-frequency, long-range coupling between prefrontal and visual cortex during attention. Science. 2009;324:1207–1210. 10.1126/science.1171402 19478185 PMC2849291

[13] Mizuseki K, Buzsaki G. Theta oscillations decrease spike synchrony in the hippocampus and entorhinal cortex. Philosophical transactions of the Royal Society of London Series B, Biological sciences. 2014;369:20120530 10.1098/rstb.2012.0530 24366139 PMC3866449

[14] Robbe D, Montgomery SM, Thome A, Rueda-Orozco PE, McNaughton BL, Buzsaki G. Cannabinoids reveal importance of spike timing coordination in hippocampal function. Nature Neuroscience. 2006;9(12):1526–1533. 10.1038/nn1801 17115043

[15] Kass RE, Kelly RC, Loh WL. Assessment of Synchrony in Multiple Neural Spike Trains Using Loglinear Point Process Models. The Annals of Applied Statistics. 2011 6;5(2B):1262–1292. 10.1214/10-AOAS429 21837263 PMC3152213

[16] Kelly RC, Kass RE. A framework for evaluating pairwise and multiway synchrony among stimulus-driven neurons. Neural Computation. 2012 8;24(8):2007–32. 10.1162/NECO_a_00307 22509967 PMC3374919

[17] Lepage KQ, Gregoriou GG, Kramer Ma, Aoi M, Gotts SJ, Eden UT, et al A procedure for testing across-condition rhythmic spike-field association change. Journal of Neuroscience Methods. 2013 2;213(1):43–62. 10.1016/j.jneumeth.2012.10.010 23164959 PMC3800189

[18] Kaufman CG, Ventura V, Kass RE. Spline-based non-parametric regression for periodic functions and its application to directional tuning of neurons. Statistics in Medicine. 2005 7;24(14):2255–65. 10.1002/sim.2104 15887309

[19] Kass RE, Eden UT, Brown EN. Analysis of Neural Data. Springer Series in Statistics; 2014.

[20] Jia X, Tanabe S, Kohn A. Gamma and the Coordination of Spiking Activity in Early Visual Cortex. Neuron. 2013 2;77(4):762–74. 10.1016/j.neuron.2012.12.036 23439127 PMC3632874

[21] Sirota A, Montgomery S, Fujisawa S, Isomura Y, Zugaro M, Buzsáki G. Entrainment of neocortical neurons and gamma oscillations by the hippocampal theta rhythm. Neuron. 2008 11;60(4):683–97. 10.1016/j.neuron.2008.09.014 19038224 PMC2640228

[22] Siapas AG, Lubenov EV, Wilson MA. Prefrontal phase locking to hippocampal theta oscillations. Neuron. 2005 4;46(1):141–51. 10.1016/j.neuron.2005.02.028 15820700

[23] Gerkin RC, Tripathy SJ, Urban NN. Origins of correlated spiking in the mammalian olfactory bulb. Proceedings of the National Academy of Sciences. 2013 9;110(42):17083–17088. 10.1073/pnas.1303830110 PMC3800994 24082089

[24] Tattersall TL, Stratton PG, Coyne TJ, Cook R, Silberstein P, Silburn Pa, et al Imagined gait modulates neuronal network dynamics in the human pedunculopontine nucleus. Nature Neuroscience. 2014 3;17(3):449–54. 10.1038/nn.3642 24487235

[25] Zelenin PV. Reticulospinal neurons controlling forward and backward swimming in the lamprey. Journal of Neurophysiology. 2011 3;105(3):1361–71. 10.1152/jn.00887.2010 21248057

[26] Lepage KQ, Kramer MA, Eden UT. The dependence of spike field coherence on expected intensity. Neural Computation. 2011 9;23(9):2209–41. 10.1162/NECO_a_00169 21671792

[27] Mitra P, Bokil H. Observed brain dynamics. Oxford University Press; 2007.

[28] Snyder AC, Smith MA. Stimulus-dependent spiking relationships with the EEG. Journal of Neurophysiology. 2015 6;. 10.1152/jn.00427.2015 PMC4556847 26108954

[29] Richardson MJE. Spike-train spectra and network response functions for non-linear integrate-and-fire neurons. Biological Cybernetics. 2008 11;99(4–5):381–92. 10.1007/s00422-008-0244-y 19011926

[30] Buzsáki G, Anastassiou CA, Koch C. The origin of extracellular fields and currents–EEG, ECoG, LFP and spikes. Nature Reviews Neuroscience. 2012;13(6):407–420. 10.1038/nrn3241 22595786 PMC4907333

[31] Grün S, Diesmann M, Aertsen A. Unitary events in multiple single-neuron spiking activity: II. Nonstationary data. Neural Computation. 2002;14:81–119. 10.1162/089976602753284464 11747535

[32] Grün S. Data-driven significance estimation for precise spike correlation. Journal of Neurophysiology. 2009;101(January 2009):1126–1140. 10.1152/jn.00093.2008 19129298 PMC2666402

[33] Pipa G, Wheeler DW, Singer W, Nikolić D. NeuroXidence: Reliable and efficient analysis of an excess or deficiency of joint-spike events. Journal of Computational Neuroscience. 2008;25:64–88. 10.1007/s10827-007-0065-3 18219568 PMC2758673

[34] Kelly RC, Kass RE, Smith Ma, Lee TS. Accounting for network effects in neuronal responses using L1 regularized point process models. Advances in Neural Information Processing Systems. 2010 1;23(2):1099–1107. 22162918 PMC3235005

[35] Park IM, Meister MLR, Huk AC, Pillow JW. Encoding and decoding in parietal cortex during sensorimotor decision-making. Nature Neuroscience. 2014;17(10):1395–1403. 10.1038/nn.3800 25174005 PMC4176983

[36] Pillow JW, Shlens J, Paninski L, Sher A, Litke AM, Chichilnisky EJ, et al Spatio-temporal correlations and visual signalling in a complete neuronal population. Nature. 2008 8;454(7207):995–9. 10.1038/nature07140 18650810 PMC2684455

[37] Koyama S, Kass RE. Spike train probability models for stimulus-driven leaky integrate-and-fire neurons. Neural Computation. 2008 7;20(7):1776–1795. 10.1162/neco.2008.06-07-540 18336078 PMC2715549

[38] Ostojic S, Brunel N. From spiking neuron models to linear-nonlinear models. PLoS Computational Biology. 2011 1;7(1):e1001056 10.1371/journal.pcbi.1001056 21283777 PMC3024256

[39] Paninski L, Brown EN, Iyengar S, Kass RE. Statistical models of spike trains In: Laing C, Lord GJ, editors. Stochastic Methods in Neuroscience. Oxford University Press; 2010 p. 272–296.

[40] Brown EN, Barbieri R, Ventura V, Kass RE, Frank LM. The time-rescaling theorem and its application to neural spike train data analysis. Neural Computation. 2002;14(2):325–346. 10.1162/08997660252741149 11802915

[41] Bosman CA, Schoffelen JM, Brunet N, Oostenveld R, Bastos AM, Womelsdorf T, et al Attentional Stimulus Selection through Selective Synchronization between Monkey Visual Areas. Neuron. 2012;75:875–888. 10.1016/j.neuron.2012.06.037 22958827 PMC3457649

[42] Buzsáki G, Draguhn A. Neuronal oscillations in cortical networks. science. 2004;304(5679):1926–1929. 10.1126/science.1099745 15218136

[43] Grothe I, Neitzel SD, Mandon S, Kreiter AK. Switching neuronal inputs by differential modulations of gamma-band phase-coherence. The Journal of Neuroscience. 2012;32(46):16172–80. 10.1523/JNEUROSCI.0890-12.2012 23152601 PMC6794021

[44] Joshua M, Adler A, Prut Y, Vaadia E, Wickens JR, Bergman H. Synchronization of Midbrain Dopaminergic Neurons Is Enhanced by Rewarding Events. Neuron. 2009;62:695–704. 10.1016/j.neuron.2009.04.026 19524528

[45] Ratté S, Hong S, De Schutter E, Prescott SA. Impact of neuronal properties on network coding: roles of spike initiation dynamics and robust synchrony transfer. Neuron. 2013 6;78(5):758–72. 10.1016/j.neuron.2013.05.030 23764282 PMC3753823

[46] Singer W. Neuronal synchrony: a versatile code for the definition of relations? Neuron. 1999;24(1):49–65. 10.1016/S0896-6273(00)80821-1 10677026

[47] Uhlhaas PJ, Singer W. Abnormal neural oscillations and synchrony in schizophrenia. Nature Reviews Neuroscience. 2010 2;11(2):100–13. 10.1038/nrn2774 20087360

[48] Womelsdorf T, Fries P. The role of neuronal synchronization in selective attention. Current Opinion in Neurobiology. 2007;17:154–160. 10.1016/j.conb.2007.02.002 17306527

[49] Stanley GB. Reading and writing the neural code. Nature Neuroscience. 2013;16:259–63. 10.1038/nn.3330 23434978

[50] Kass R, Ventura V. A spike-train probability model. Neural Computation. 2001;1720:1713–1720. 10.1162/08997660152469314 11506667

[51] Graves S, Hooker G, Ramsay J. Functional data analysis with R and MATLAB. Springer, New York; 2009.

[52] Burton SD, Urban NN. Greater excitability and firing irregularity of tufted cells underlies distinct afferent-evoked activity of olfactory bulb mitral and tufted cells. The Journal of Physiology. 2014;00:1–22. 10.1113/jphysiol.2013.269886 PMC4227897 24614745

[53] Feng G, Mellor RH, Bernstein M, Keller-Peck C, Nguyen QT, Wallace M, et al Imaging neuronal subsets in transgenic mice expressing multiple spectral variants of GFP. Neuron. 2000;28:41–51. 10.1016/S0896-6273(00)00084-2 11086982

[54] Snyder AC, Morais MJ, Willis CM, Smith MA. Global network influences on local functional connectivity. Nature Neuroscience. 2015 05;18(5):736–743. 10.1038/nn.3979 25799040 PMC4641678

[55] Smith MA, Sommer MA. Spatial and temporal scales of neuronal correlation in visual area V4. The Journal of Neuroscience. 2013;33:5422–32. 10.1523/JNEUROSCI.4782-12.2013 23516307 PMC3712790

[56] Shoham S, Fellows MR, Normann RA. Robust, automatic spike sorting using mixtures of multivariate t-distributions. Journal of Neuroscience Methods. 2003 8;127(2):111–122. 10.1016/S0165-0270(03)00120-1 12906941

[57] Kelly RC, Smith MA, Samonds JM, Kohn A, Bonds AB, Movshon JA, et al Comparison of recordings from microelectrode arrays and single electrodes in the visual cortex. The Journal of Neuroscience. 2007;27:261–264. 10.1523/JNEUROSCI.4906-06.2007 17215384 PMC3039847

[58] Ray S, Maunsell JHR. Different origins of gamma rhythm and high-gamma activity in macaque visual cortex. PLoS Biology. 2011;9(4). 10.1371/journal.pbio.1000610 21532743 PMC3075230

